# Azacitidine and donor lymphocyte infusion for patients with relapsed acute myeloid leukemia and myelodysplastic syndromes after allogeneic hematopoietic stem cell transplantation: A meta-analysis

**DOI:** 10.3389/fonc.2022.949534

**Published:** 2022-08-05

**Authors:** Xuefeng Li, Wen Wang, Xin Zhang, Yu Wu

**Affiliations:** ^1^ Department of Hematology and Institute of Hematology, West China Hospital, Sichuan University, Chengdu, China; ^2^ Chinese Evidence-based Medicine Center and Cochrane China Center, West China Hospital, Sichuan University, Chengdu, China

**Keywords:** azacitidine, donor lymphocyte infusion, acute myeloid leukemia, myelodysplastic syndromes, relapse

## Abstract

**Background:**

For patients with relapsed acute myeloid leukemia (AML) and myelodysplastic syndromes (MDS) after allogeneic hematopoietic stem cell transplantation (allo-HSCT), azacitidine with donor lymphocyte infusion (DLI) is a feasible option to perform a preemptive or salvage treatment. However, its efficacy lacked comprehensive analysis, and this study aimed to fill this gap.

**Methods:**

We searched potential studies in PUBMED, EMBASE, and the Cochrane Central Register of Controlled Trials. Thirteen studies involving 811 patients were analyzed. The inverse variance method was used to calculate the pooled proportion and 95% confidence interval (CI). Subgroup analysis was performed to explore the source of heterogeneity.

**Results:**

The rate of pooled complete remission + partial remission (CR + PR), CR, and 2-year overall survival (OS) were 30% (95% CI: 22%–39%), 21% (95% CI: 16%–28%), and 31% (95% CI: 27%–35%), respectively. The pooled acute graft-versus-host disease (GvHD) and chronic GvHD rates were 15% (95% CI: 9%–23%) and 14% (95% CI: 8%–23%), respectively. Adverse cytogenetics and a higher percentage of bone marrow (BM) blasts at relapse were correlated with worse CR + PR and CR (interaction p < 0.05). Higher 2-year OS was found in patients with lower BM blasts at relapse or a longer time from allo-HSCT to relapse (interaction p < 0.05). Furthermore, the preemptive treatment for molecular relapse/minimal residual disease positivity resulted in much better outcomes than that for hematological relapse, both in terms of CR and 2-year OS (interaction p < 0.001).

**Conclusion:**

The regimen of azacitidine and DLI could safely improve the outcomes of relapsed AML/MDS after allo-HSCT, especially in those with signs of early relapse. The administration of targeted medicines in azacitidine-based therapies may further improve the outcomes of relapsed AML/MDS.

## Introduction

Allogeneic hematopoietic stem cell transplantation (allo-HSCT) has become a widely used therapy in acute myeloid leukemia (AML) and myelodysplastic syndromes (MDS) (1, 2). However, relapse after allo-HSCT often indicates poor outcomes for these patients (3). Currently available treatments for relapsed patients include re-induction chemotherapy, donor lymphocyte infusion (DLI), second allo-HSCT, targeted medicine-based therapies, and attending clinical trials (4). Nevertheless, intensive chemotherapy or the second allo-HSCT may not be suitable for old or frail patients, owing to a high-risk of severe adverse effects. Therefore, alternative methods need to be explored to improve outcomes.

Azacitidine, as a DNA methyltransferase inhibitor, has been commonly used in various hematological diseases, such as high-risk MDS, chronic myelomonocytic leukemia (CMML), and AML (5, 6). Hypomethylating agents can promote the expression of tumor testis antigens on both AML and MDS cells and induce CD8^+^ T cells to recognize silenced tumor-associated antigens (7, 8), ultimately exerting antitumor effects in myeloid neoplasms. Meanwhile, previous studies have revealed that relapsed myeloid neoplasm after allo-HSCT could express a higher level of gene methylation (9, 10), which indicates that azacitidine may play a unique role in the treatment of those patients.

DLI is a simple and effective therapy for relapsed myeloid neoplasms after allografting since 1993 (11, 12). In previous studies, DLI was found to strengthen the anti-leukemic T cells and reverse T-cell exhaustion, through increased IFN-γ and reduced T-cell inhibitory receptors (13). While DLI may enhance the graft-versus-leukemia (GVL) effect and extend the survival of patients (14), the incidence of severe acute graft-versus-host disease (GvHD) after DLI administration could limit the use of it and even cause treatment-related mortalities (15). Interestingly, azacitidine was proven to mitigate GvHD in both murine preclinical transplant models and human clinical trials, with effective GVL remained (16–18). Therefore, azacitidine with DLI may be an effective choice for post-HSCT relapse.

There have been studies on the efficacy of using azacitidine and DLI as salvage or preemptive treatment for relapsed AML or MDS after allo-HSCT. Some studies have attempted to combine subsequent second allo-HSCT or other medical protocols to construct variable therapeutic regimens (4). However, most of the studies were small sized and lacked comprehensive statistical analysis. Furthermore, the factors contributing to the efficacy remained unclear. Therefore, this meta-analysis aimed to provide evidence-based information for clinicians to solve the abovementioned questions.

## Materials and methods

In this study, we followed the standards set by Meta-analysis of Observational Studies in Epidemiology (19).

### Eligibility criteria

We planned to include single-arm studies that evaluate the treatment effects of azacitidine among patients with relapsed AML and MDS after allo-HSCT, and DLI was the only additional optional intervention. The prespecified outcomes included complete remission (CR), partial remission (PR), and 2-year overall survival (OS) rates. Studies in which a subsequent allo-HSCT after azacitidine and DLI regimen was performed or had less than 10 patients were excluded.

### Literature search

We searched PUBMED, EMBASE, and Cochrane Controlled Register of Trials (CENTRAL) from the study inception to 7 December 2021. We combined Medical Subject Headings terms and free-text terms to search for potential target studies (Supplementary Text). Moreover, we reviewed the reference lists of the included studies to identify additional studies.

### Article quality assessment

The methodological quality of each study was assessed *via* the methodological index for non-randomized studies (MINORS) guidelines (20). MINORS has 12 items, of which 8 apply to both non-comparative and comparative studies, whereas the remaining 4 are exclusively applied to comparative studies. The eight items applicable for both non-comparative and comparative studies include: study aims, consecutive patient inclusion criteria, prospective pooling of data, endpoint consistent with the study aim, unbiased evaluation of endpoints, follow-up period, loss to follow-up less than 5%, and prospective calculation of the sample size. The items were scored 0 (not reported), 1 (reported but inadequate), or 2 (reported and adequate), and the total score represented the summary assessment of the bias risk for each study.

### Definition and treatment

Hematological relapse was defined as a morphological occurrence in the bone marrow (BM) (blasts >5%) and the detection of mixed chimerism, regardless of extramedullary disease. The reappearance of dysplastic features fulfilling the diagnosis criteria for MDS was also defined as hematological relapse in a study (21). Molecular relapse was defined as the recurrence of disease-specific markers (i.e., recurrent fusion gene, abnormal karyotype, and/or gene mutation) in the blood or BM, with blasts <5% in the bone marrow. Minimal residual disease positivity (MRD^+^) was evaluated by using qPCR or flow cytometry. Complete remission with or without incomplete recovery (CR/CRi) was a combination of “complete remission (CR)” and “CR with incomplete hematological recovery (CRi).” The definitions of CR, CRi, CR without minimal residual disease (CRm), and partial response (PR) for AML were based on the recommendation from the European LeukemiaNet (22); CR and PR for MDS were based on the clinical application and proposal for the modification of the International Working Group response criteria in myelodysplasia (23). The cytogenetic risk classification for AML was based on the recommendation from the European LeukemiaNet. The International Prognostic Scoring System was used to evaluate the cytogenetic risk for MDS (22, 24).

The included studies varied in conditioning regimens, which comprised standard-dose myeloablative conditioning (MAC), reduced-intensity conditioning (RIC), and non-myeloablative conditioning (NMAC). However, most of the studies did not present the details of conditioning regimens; instead, these studies only presented the number of patients who accepted MAC, RIC, or NMAC. The proportion of MAC in each study ranged from 13.3% to 90.9%.

In general, azacitidine was administered at a dose ranging from 50 to 100 mg/m^2^ for 5–7 consecutive days every month or 28 days. Two studies used a low-dose regimen of 100-mg azacitidine per day for 3 consecutive days every 21 days. Patients received an average of two-to-six cycles of azacitidine. DLI administration was restrained by the patients’ general conditions and disease status; therefore, the proportion of DLI administration in each study ranged from 40% to 100%. In a monthly or 28- day schedule of azacitidine, DLI was generally administered at a dose ranging from 3 × 10^5^ to 5 × 10^8^ CD3^+^/kg on day 8 of every second cycle. In the low-dose regimen of azacitidine, DLI was generally used on day 10 of every cycle at a dose of 3 × 10^5^ to 2 × 10^6^ CD3^+^/kg. Meanwhile, part of the patients in one study received DLI after the failure of azacitidine (25). Patients received an average of one-to-two cycles of DLI in these studies.

### Statistical analysis

In this study, we used the Metaprop module in the R-4.0.5 statistical software package to analyze the efficacy of this regimen in relapsed AML/MDS. The inverse variance method was used to calculate the pooled proportion and 95% confidence intervals (CIs). Heterogeneity was calculated by the chi-squared test (χ^2^ test) and I-squared test (I^2^ test). The results were based on the random-effect model when heterogeneity was present (I^2^ > 50%); otherwise, the fixed-effect model would be chosen.

Studies in this analysis were all single arm and lacked control groups. Since some of the included studies were retrospective and lacked complete information on baseline characteristics or outcomes, we did not conduct a multivariate regression analysis.

We performed seven subgroup analyses to explore the sources of heterogeneity: age (≤55 vs. >55 years old), the proportion of adverse cytogenetics (≤40% vs. >40%), the percentage of BM blasts at relapse (≤20% vs. >20%), the proportion of myeloablative conditioning at the latest HSCT (≤40% vs. >40%), the proportion of CR patients at HSCT (≤40% vs. >40%), and the time from HSCT to the latest relapse (<6 months vs. >6 months). Moreover, the outcomes based on different relapse types (hematological relapse vs. molecular relapse/MRD^+^) were compared. We set the cutoff value of age according to previously published clinical trials. We set the cutoff proportion to assess whether a cytogenetic risk or the disease status at HSCT would result in different outcomes. The cutoff value of the interval time from HSCT to relapse was based on the available data of outcomes in the included studies. Meanwhile, balancing the number of studies or patients in each group was also a consideration for setting these cutoff values. Interaction p-values <0.05 were considered statistically significant. We performed subgroup analysis only if there were at least two studies in each subgroup category. Sensitivity analysis was performed to assess the effect with the removal of the largest sample size among all studies.

## Results

### Literature search results

**Figure 1:**
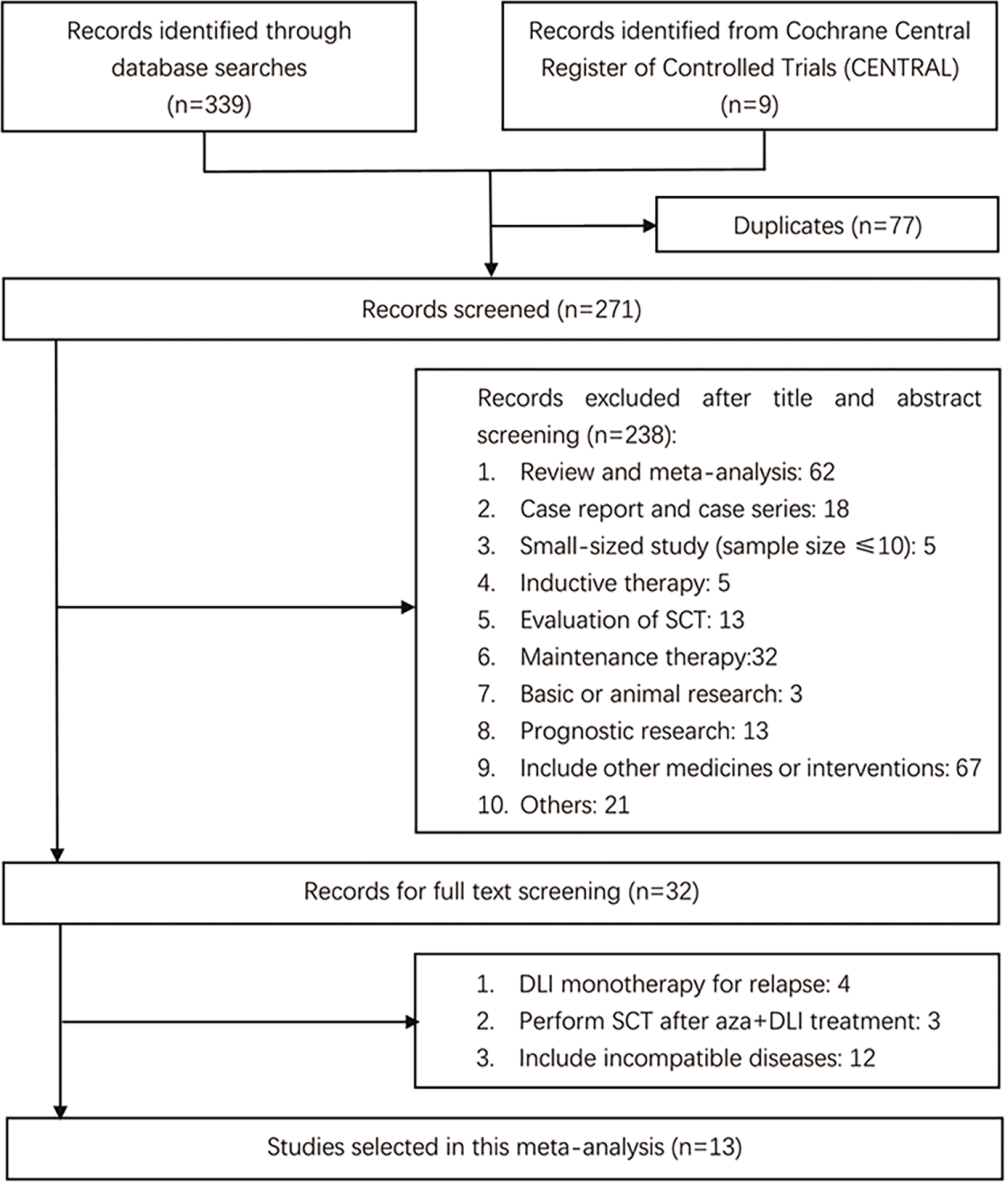
Literature search process.

The process of literature search is presented in **Figure 1**. After excluding duplicates and those that did not meet the eligibility criteria, we ultimately included 13 studies involving 811 patients to perform our meta-analysis (21, 25–36).

The characteristics of the included studies and patients are illustrated in **Tables 1**, **2**. Of 13 included studies, 10 were retrospective single-arm and 3 were phase II prospective single-arm studies. The 13 studies involved 16–157 participants, with the number of DLI administration ranging from 12 to 105, the mean age ranging from 50 to 62 years, and the average or maximal time from the commencement of azacitidine to relapse ranging from 4 to 36 days. The median time of achievement to the best response (CR or PR) in the included studies ranged from 79 to 141 days. The proportion of CR + PR, CR, PR, and 2-year OS among the included studies ranged from 10% to 55%, 8% to 41%, 0 to 23%, and 16% to 41%, respectively (**Table 3**).

**Table 1:** Characteristics of the included studies.

No.	Study	Design	Registration number	Country	No. of patients	No. of DLIs
1	Aydin et al. (2021) (26)	Retrospective, single-arm	–	Italy	27	12
2	Claiborne et al. (2019) (27)	Retrospective, single-arm	–	USA	28	28
3	Craddock et al. (2016) (25)	Retrospective, single-arm	–	Europe	157/181^a^	Allowed
4	Czibere et al. (2010) (28)	Retrospective, single-arm	–	Germany	22	18
5	Liberatore et al. (2020) (29)	Retrospective, single-arm	–	Italy	40	16
6	Lübbert et al. (2010) (30)	Retrospective, single-arm	–	Germany	26	17
7	Martinez-Cibrian et al. (2017) (31)	Retrospective, single-arm	–	UK	16	16
8	Poiré et al. (2021) (32)	Phase II prospective, single-arm	–	Belgium	49	49
9	Rautenberg et al. (2020) (33)	Retrospective, single-arm	–	Germany	151	105
10	Schroeder et al. (2013) (34)	Phase II prospective, single-arm	NCT00795548	Germany	30	25
11	Schroeder et al. (2015) (21)	Retrospective, single-arm	–	Germany	154	104
12	Steinmann et al. (2015) (35)	Retrospective, single-arm	–	Germany	72	72
13	Woo et al. (2017) (36)	Phase II prospective, single-arm	NCI01083706	USA	39	Allowed

a: A total of 24 patients in Craddock et al. (2016) (25) were excluded since they were allografted before an assessment of response to azacitidine salvage was made. Therefore, only 157 patients were included in this meta-analysis.

**Table 2:** Baseline characteristics of the included patients^a.^.

Study	AML/MDS/others^b^	Average age	Baseline cytogenetic risk			Status at HSCT	No. of MAC	Type of relapse			BM blasts at relapse
			Favorable	Intermediate	Adverse			h-Re	m-Re	MRD^+^	
Aydin et al. (2021) (26)	21/6/0	–	–			–	–	26	1	0	–
Claiborne et al. (2019) (27)	19/9/0	57	1	16	7	1st CR: 13, >1st CR: 6, PR: 1, MRD^+^: 4, PD: 1, NR or SD: 1.	–	20	8	0	6%
Czibere et al. (2010) (28)	13/8/1	50	1	17	4	–	20	22	0	0	–
Liberatore et al. (2020) (29)	40/0/0	–	–			–	–	26	0	14	–
Lübbert et al. (2010) (30)	24/2/0	62	2	9	11	–	–	26	0	0	59%
Martinez-Cibrian et al. (2017) (31)	12/4/0	60	–			1st CR: 8; 2nd CR: 2; PR: 2, untreated: 4.	–	–			–
Poiré et al. (2021) (32)	30/19/0	60	0	22	25	1st CR: 39, 2nd CR: 4, PR: 1, untreated: 3.	38	–			10%
Rautenberg et al. (2020) (33)	90/49/12	54	–			1st CR: 45, 2nd CR: 5, PR: 15, no remission: 39, untreated: 43, missing: 4.	54	92	59	0	7%
Schroeder et al. (2013) (34)	28/1/1	55	2	13	14	1st CR: 12, 2nd CR: 2, no remission: 16.	4	30	0	0	34%
Schroeder et al. (2015) (21)	124/28/2	55	21	76	55	CR: 59, relapse: 32 induction failure: 26, untreated: 32, missing: 5.	64	135	19	0	13%
Steinmann et al. (2015) (35)	67/5/0	62	4	24	30	1st CR: 8, 2nd CR: 5, relapse: 19, untreated: 12, primary refractory: 28.	12	72	0	0	25%
Woo et al. (2017) (36)	26/13/0	52	0	16	23	–	–	5	34	0	–

AML, acute myeloid leukemia; MDS, myelodysplastic syndrome; HSCT, stem cell transplantation; DLI, donor lymphocyte infusion; BM, bone marrow; CR, complete remission; PR, partial remission; MAC, myeloablative conditioning; h-Re, hematological relapse; m-Re, molecular relapse; MRD^+^, minimal residual disease positivity; PD, progressive disease; NR, no response; SD, stable disease.

a: Since 24 patients in Craddock et al. (2016) (25)were excluded in this meta-analysis, the baseline characteristics of the included patients were unavailable; b: several studies included a few patients diagnosed as chronic myelomonocytic leukemia, Ph- chronic myelomonocytic leukemia, or MDS/MPS unclassifiable.

**Table 3:** Outcomes of the azacitidine and donor lymphocyte infusion regimen.

Study	Median survival	CR+PR rate	CR rate		PR rate	2-year OS	Incidence of aGvHD	Incidence of cGvHD
			CR/CRi	CRm				
Aydin et al. (2021) (26)	–	–	22%	–	–	–	–	–
Claiborne et al. (2019) (27)	10 months	–	36% (total), 50% (m-Re), 28% (h-Re)	32%	–	35%	11%	36%
Craddock et al. (2016) (25)	–	29%^a^	15%	–	14%	–	–	–
Czibere et al. (2010) (28)	144 days	41%	23%	–	18%	23%	33%	18%
Liberatore et al. (2020) (29)	10 months	55% (total), 57% (MRD^+^), 54% (h-Re)	33% (total), 43% (MRD^+^), 27% (h-Re)	–	–	41% (total), 77% (MRD^+^), 22% (h-Re)	8%	13%
Lübbert et al. (2010) (30)	136 days	16%	16%	–	0	16%	8%	4%
Martinez–Cibrian et al. (2017) (31)	–	–	13%	–	–	–	–	–
Poiré et al. (2021) (32)	6 months	22%	20%	–	2%	–	–	13%
Rautenberg et al. (2020) (33)	–	46%	41% (total), 61% (m-Re), 28% (h-Re). 52% (time from HSCT to relapse > 6 months), 33% (< 6 months)	–	5%	38% (total), 55% (m-Re), 29% (h-Re). 51% (time from HSCT to relapse > 6 months), 30% (< 6 months)	–	–
Schroeder et al. (2013) (34)	–	30%	23%	–	7%	17%	37%	17%
Schroeder et al. (2015) (21)	–	33%	27% (total), 68% (m-Re), 21% (h-Re). 31% (time from HSCT to relapse > 6 months), 22% (< 6 months)	–	6%	29% (total), 62% (m-Re), 25% (h-Re). 39% (time from HSCT to relapse > 6 months), 19% (< 6 months)	23%	27%
Steinmann et al. (2015) (35)	108 days	10%	10%	–	0	–	10%	4%
Woo et al. (2017) (36)	–	31%	8%	–	23%	25%	8%	–

aGvHD, acute graft-versus-host disease; cGvHD, chronic graft-versus-host disease; h-Re, hematological relapse; m-Re, molecular relapse; MRD^+^, minimal residual disease positivity.

a: Since 24 patients were excluded in this meta-analysis, only the CR and PR rate were available for included patients in Craddock et al. (2016) (25).

### Assessment of article quality

The methodological quality of included studies is summarized in **Supplementary Table 1**. In this meta-analysis, all included studies were single-arm studies; therefore, we only used the first eight items to assess the methodological quality. Among 13 included studies, the total score ranged from 9 to 12.

### Efficacy of azacitidine and donor lymphocyte infusion regimen

**Figure 2:**
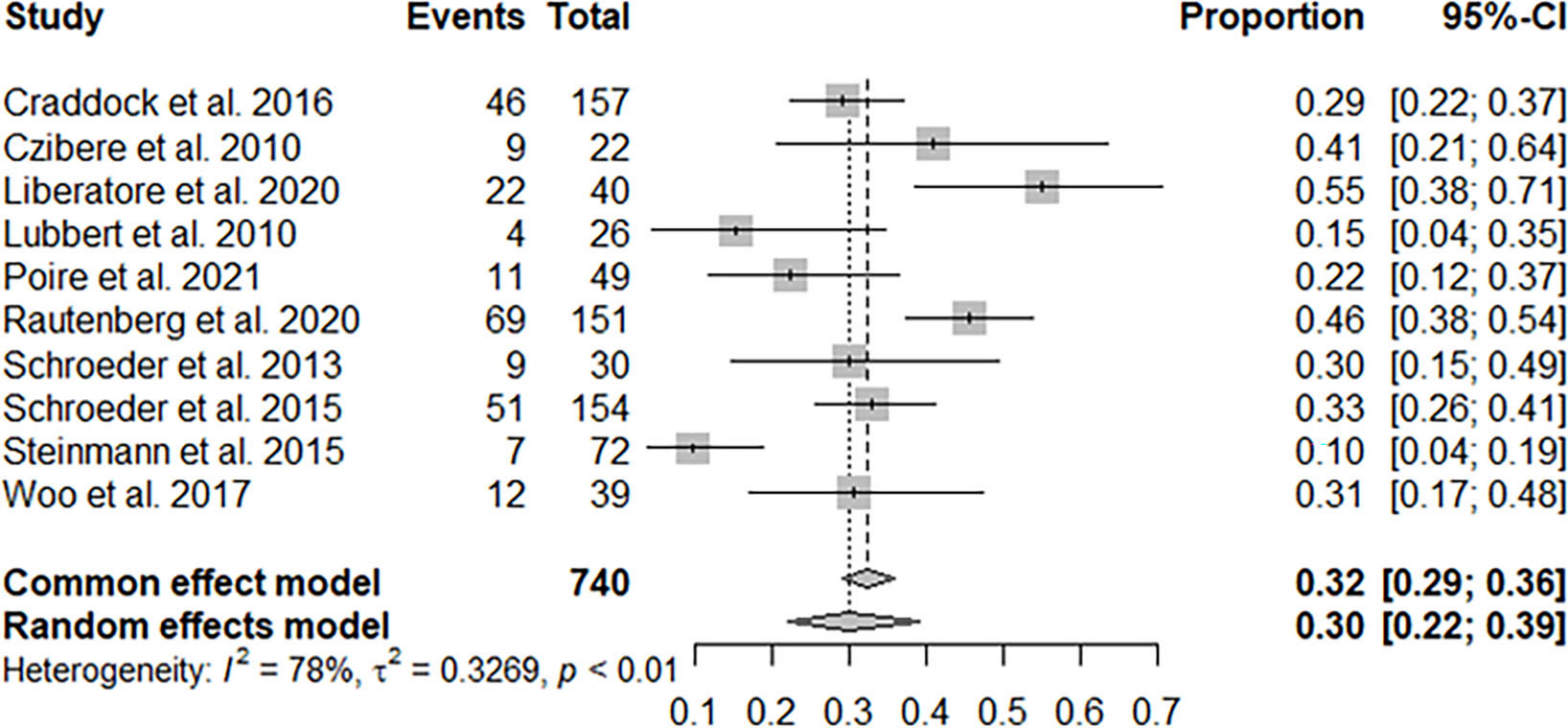
Pooled complete remission (CR) + partial remission of azacitidine and donor lymphocyte infusion (DLI) regimen.

**Figure 3:**
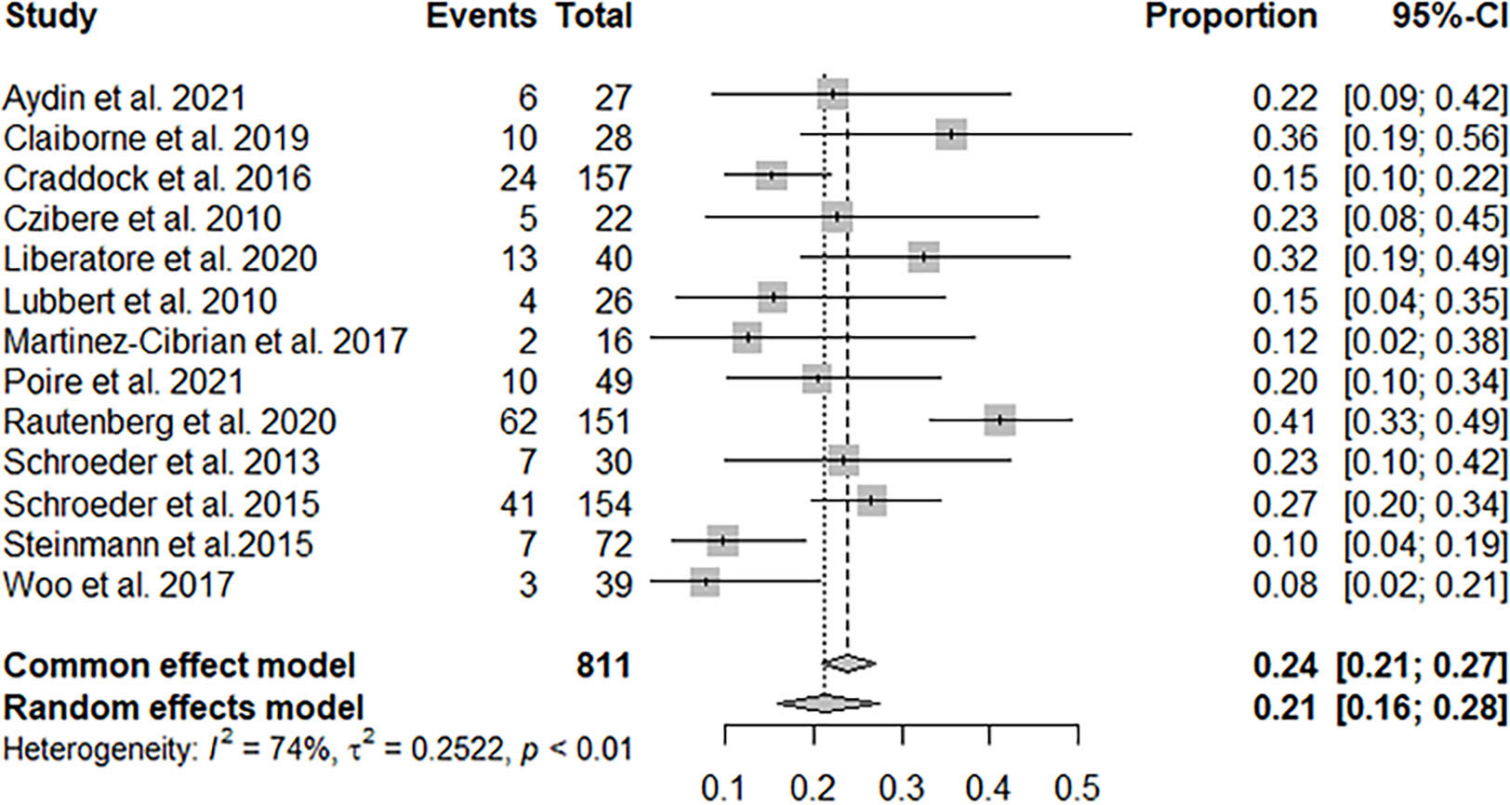
Pooled CR of azacitidine and DLI regimen.

**Figure 4:**
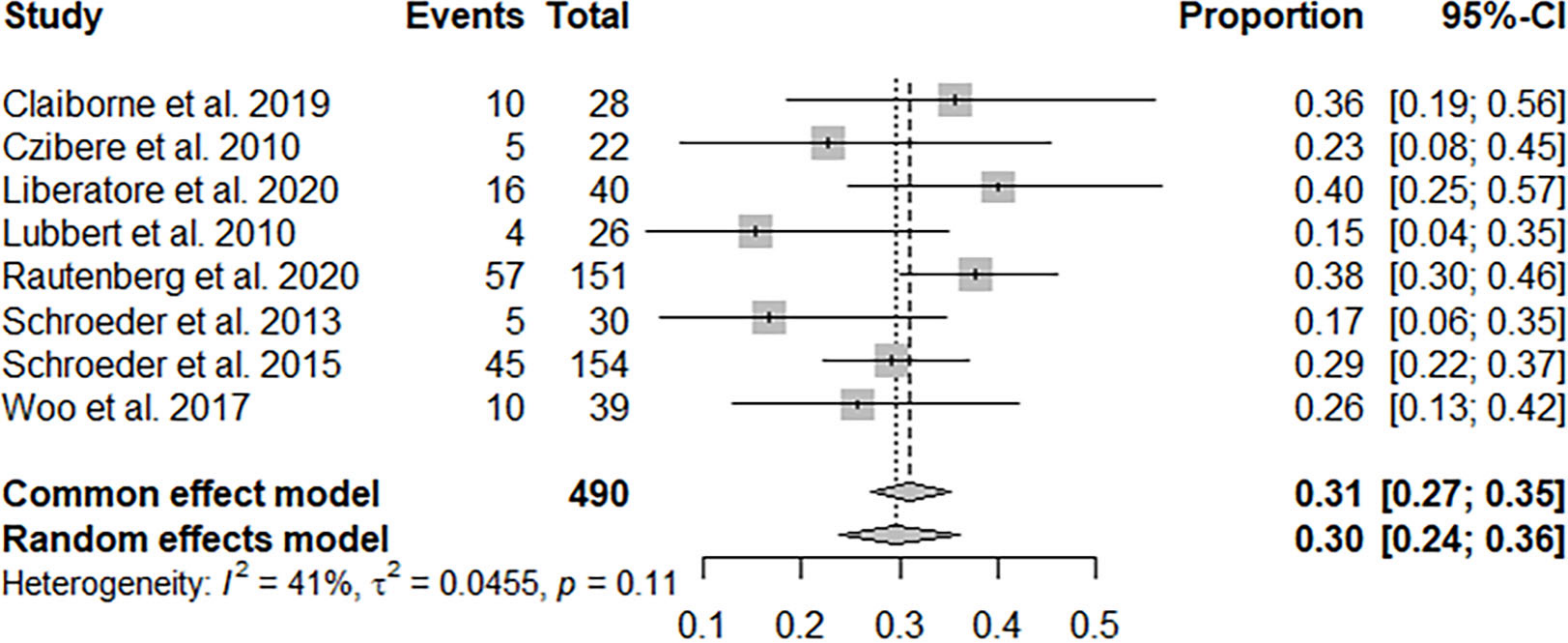
Pooled 2-year overall survival of azacitidine and DLI regimen.

Ten studies with 740 patients reported CR + PR, and the pooled CR + PR rate was 30% (95% CI: 22%–39%; **Figure 2**). Thirteen studies with 811 patients reported CR, and the pooled CR rate was 21% (95% CI: 16%–28%; **Figure 3**). Eight studies with 490 patients reported 2-year OS, and the pooled 2-year OS rate was 31% (95% CI: 27%–35%; **Figure 4**).

### Subgroup analysis of complete remission + partial remission, complete remission, and 2-year overall survival

Subgroup analysis (**Table 4**) showed that patients with bone marrow (BM) blasts ≤20% at relapse had higher CR + PR, CR, and 2-year OS than those with BM blasts >20% (interaction p < 0.05). Cytogenetic risk could also affect the outcomes of CR + PR and CR (interaction p < 0.05). CR + PR was found to be higher in patients aged ≤55 years than those aged >55 years (interaction p < 0.001). And better 2-year OS was observed in the group of longer time from the latest HSCT to relapse (>6 months) (interaction p < 0.05). Furthermore, the preemptive treatment for the molecular relapse/MRD^+^ group resulted in much better outcomes than that for the hematological relapse group, both in CR and 2-year OS (both interaction p < 0.001). In the subgroup analysis by status at HSCT and conditioning of HSCT, they showed no statistical significance (interaction p > 0.05).

**Table 4:** Subgroup analysis of complete remission (CR) + partial remission, CR, and 2-year overall survival.

Subgroup	CR + PR rate (95% CI)	Interaction *p*-value	CR rate (95% CI)	Interaction *p*-value	2-year OS rate (95% CI)	Interaction *p*-value
**Age, year**						
≤55	0.371 (0.307–0.440)	<0.001	0.241 (0.150–0.365)	0.325	0.308 (0.265–0.355)	0.464
>55	0.150 (0.093–0.232)		0.175 (0.109–0.269)		0.259 (0.160–0.392)	
**Adverse cytogenetics (%)**						
≤40%	0.343 (0.281–0.411)	0.013	0.270 (0.213–0.335)	0.004	0.294 (0.236–0.360)	0.088
>40%	0.202 (0.134–0.293)		0.144 (0.098–0.205)		0.200 (0.131–0.292)	
**Proportion of CR patients at HSCT (%)**						
≤40%	0.275 (0.126–0.499)	0.679	0.248 (0.119–0.445)	0.897	0.358 (0.265–0.462)	0.256
>40%	0.232 (0.158–0.327)		0.236 (0.169–0.319)		0.256 (0.146–0.410)	
**Conditioning of HSCT (%)**						
MAC ≤40%	0.258 (0.113–0.486)	0.593	0.227 (0.104–0.424)	0.804	–	
MAC >40%	0.316 (0.258–0.379)		0.249 (0.197–0.310)		–	
**BM blasts at relapse (%)**						
≤20%	0.346 (0.250–0.458)	0.021	0.312 (0.237–0.399)	0.004	0.336 (0.285–0.392)	0.011
>20%	0.163 (0.088–0.284)		0.143 (0.087–0.225)		0.161 (0.086–0.281)	
**Time from relapse to HSCT (months)**						
<6	–		0.280 (0.214–0.357)	0.112	0.245 (0.175–0.330)	0.001
>6	–		0.406 (0.273–0.554)		0.443 (0.360–0.529)	
**Type of relapse**						
Hematological	–		0.211 (0.164–0.267)	<0.001	0.245 (0.201–0.294)	<0.001
Molecular or MRD^+^	–		0.590 (0.491–0.682)		0.598 (0.495–0.693)

CR, complete remission; PR, partial remission; OS, overall survival; BM, bone marrow; MAC, myeloablative conditioning; MRD^+^, minimal residual disease positivity.

### Graft-versus-host disease rate of the azacitidine and donor lymphocyte infusion regimen

Eight studies with 411 patients reported the acute GvHD rate after the azacitidine regimen initiation, and the pooled acute GvHD rate was 15% (95% CI: 9%–23%). Eight studies with 421 patients reported the chronic GvHD rate, and the pooled chronic GvHD rate was 14% (95% CI: 8%–23%). Four studies with 251 patients reported the incidence of grade III/IV acute GvHD during the azacitidine and DLI treatment, and the pooled incidence was 9% (95% CI: 6%–13%) (21, 27, 34, 36).

### Adverse events of azacitidine and donor lymphocyte infusion regimen

The records of adverse events in the included studies were generally incomplete. According to some contents described in these studies, skin reactions and gastrointestinal symptoms were prone to occur during the treatment process, and they were mostly mild. However, according to a prospective study, grade III/IV neutropenia, thrombopenia, and anemia were observed in 65%, 63%, and 33% patients during the treatment process and 33% of the patients had undergone grade III/IV infections (34). Meanwhile, one study reported that 39% of patients were readmitted at least once because of infectious complications; 15 patients (21%) were associated with grade III/IV neutropenia, and 2 of them were fatal (35). Therefore, the treatment process could be suspended or terminated due to severe GvHD, serious infections, and other hematological or non-hematological events.

### Sensitivity analysis

When we removed the study with the largest sample size, the pooled CR + PR, CR, and 2-year OS remained stable (interaction p = 0.998, interaction p = 0.862, and interaction p = 0.897, respectively). We performed two additional comparisons to evaluate the influence of the proportion of DLI administration on our conclusions, and the differences were all statistically insignificant (interaction p > 0.05) (**Supplementary Table 2**).

## Discussion

AML/MDS relapse following allograft typically indicates a poor prognosis. The 1- and 3-year OS of relapsed AML after allo-HSCT were only 22% and <10%, respectively (37). The long-term survival rate of relapsed AML after allo-HSCT was reported to be only 5% (38).

In this meta-analysis for azacitidine and DLI regimen, we found that the younger group (≤55 years old) had a better performance in CR + PR (interaction p < 0.001) than the older one. However, myeloablative conditioning and status at HSCT (proportion of CR) did not show remarkable impacts on the ultimate CR + PR, CR, or 2-year OS. Meanwhile, several studies have reported that relapsed MDS patients who received azacitidine and DLI have resulted in better 2-year OS than AML patients (p < 0.05), but differences in CR were insignificant (p > 0.05) (21, 27, 33).

Although DLI is widely used in relapsed myeloid neoplasms following allograft, two studies, including one prospective study and one large-scale retrospective study, claimed that DLI administration did not improve the CR or 2-year OS in azacitidine-based treatment (25, 32). The relative ineffectiveness of DLI in AML may be explained by the massive tumor burden, the possibility of the downregulation of HLA Class II on leukemic blasts, and the development of immune evasion (39–41). Various doses or schedules of DLI may result in outcome variation. For instance, one of the included studies reported that the responders received more cycles of DLI than those not achieving remission (2.9 vs. 1.7 cycles, p = 0.024) (27). Some researchers reported the efficacy and potential mechanism of the rapid taper of immunosuppression in treating relapsed hematological malignancies, which may enhance the effectiveness of DLI by promoting the immune reconstruction or restoring the antitumor function of T lymphocytes (42). In general, more rigorous RCTs are required to prove the effectiveness of DLI or DLI combined with the rapid taper of immunosuppression for patients with relapsed AML/MDS.

Our meta-analysis suggests that a cytogenetic risk could play a role in the outcomes of azacitidine and DLI regimen. It is widely acknowledged that adverse cytogenetics is associated with a higher relapse/refractory rate and lower remission rate (43, 44). Similarly, CR + PR and CR statistically decrease in studies with higher proportions of adverse cytogenetics (40%) in our analysis (interaction p = 0.013 and interaction p = 0.004, respectively). In addition, as mentioned in some previous studies (21, 33, 45), our study showed that longer time from the latest HSCT to relapse (> 6 months) indicated a better 2-year OS (interaction p < 0.05).

According to our study, high BM blasts (20%) at relapse was associated with a much lower CR + PR, CR, and 2-year OS during the treatment process (all interactions p < 0.05). Therefore, clinicians need to take other therapies into consideration when dealing with patients who have a high BM blast percentage at relapse. Furthermore, our meta-analysis confirmed the significant differences in the outcomes of relapse types (hematological relapse vs. molecular relapse/MRD^+^), and the azacitidine and DLI regimen achieved much better performance in the preemptive treatment group (CR, 21.1% vs. 59.0%; 2-year OS, 24.5% vs. 59.8%, both interactions p < 0.001). In a recent study concerning the preemptive or salvage treatment for relapsed AML, researchers found that early MRD-driven interventions improved the patient’s outcomes compared with morphological occurrence, which resulted in 57.0% vs. 7.0% (p = 0.01) on the 2-year progression-free survival (PFS), and 77.0% versus 22.0% (p = 0.01) on the 2-year OS (29). In addition, a previous study also mentioned the efficacy of preemptive DLI for AML/MDS patients with persisting or declining mixed donor/recipient chimerism after HSCT, which partly demonstrated the effectiveness of DLI in a limited tumor burden (46). These results highlight the importance of consistent disease monitoring and early initiation of interventions. MRD-driven preemptive therapies might be helpful for those who belong to the adverse cytogenetic group or high-risk group.

Except for DLI, some researchers had attempted to combine lenalidomide with hypomethylating agents to treat patients with relapse; however, the results did not show remarkable advantages compared with azacitidine and DLI regimen (47). In recent years, targeted therapies indicated a promising prospect in the treatment of refractory/relapsed AML/MDS. At the time of relapse, it is important to immediately perform a mutational screening and cytogenetic analysis since the clonal evolution of disease is frequent (48). For refractory/relapsed AML patients with isocitrate dehydrogenase (IDH) 1/2 mutation, IDH1 inhibitors, such as ivosidenib, and IDH2 inhibitors, such as enasidenib, have presented well-tolerated outcomes in clinical trials (49, 50). For patients with FMS-like tyrosine kinase 3 (FLT3) mutations, gilteritinib, an FLT3 inhibitor, improved the patients’ outcomes (51, 52). Venetoclax has been approved for the treatment of older or unfit AML patients with a higher response rate compared with traditional therapy, but further clinical trials are needed to verify its role in refractory/relapsed AML postallo-HSCT (53, 54). Relapsed and newly diagnosed patients with AML had a significantly higher percentage of CD8^+^ T cells with PD-1 expression in the BM (55). Recently, several trials had disclosed the outcomes of azacitidine with PD-1 antibodies as a salvage therapy for refractory/relapsed AML. The use of avelumab with azacitidine for refractory/relapsed AML achieved only a CR of 10.5% (56); in another study, the combination of nivolumab and azacitidine resulted in a CR of 22% (57).

Although we did not include studies in which post-HSCT relapsed AML/MDS patients received azacitidine and DLI, then bridged to subsequent-HSCT, there are studies that evaluated the efficacy of azacitidine with DLI as a bridging treatment to subsequent allo-HSCT in AML/MDS (58, 59). These studies reported better OS and/or PFS in patients who received a subsequent allo-HSCT than that in patients who received only azacitidine with DLI. These results indicated that subsequent curative therapies may further improve the survival of relapsed patients after the azacitidine treatment, and RCTs are needed to evaluate the potential additional effect of azacitidine-based therapies as a bridge treatment.

Our study is the first meta-analysis to show that azacitidine-based therapies are feasible options for older (mean age ranging from 52 to 62 years) and frail patients with relapsed AML/MDS after allo-HSCT; when combined with DLI, a preemptive strategy is prior to salvage treatment. However, our study has some limitations. Due to ethical and analytical considerations, studies in this analysis were single arm and lacked control groups. Some of the studies selected in our meta-analysis were retrospective and lacked complete information on baseline characteristics or outcomes, so we did not conduct a multivariate regression analysis. Moreover, since the time span between these studies was relatively long, pre-HSCT therapies and conditioning regimens at HSCT might be different; consequently, heterogeneity does exist. Therefore, our results require confirmation by large-sized RCTs. There is a need to explore more effective regimens as salvage treatment to further improve the prognosis of patients with relapsed AML/MDS.

## Data availability statement

The original contributions presented in the study are included in the article/**Supplementary Material**. Further inquiries can be directed to the corresponding author.

## Author contributions

XL collected and analyzed the data and wrote the article. WW provided methodological guidance, helped in subgroup analysis, and prepared the figures. XZ reviewed the data and helped in preparing the tables. YW designed research, provided the plan, and modified the article. All authors read and approved the final manuscript.

## Funding

This research received Sichuan Provincial Academic and Technical Support 22ZDYF2091.

## Conflict of interest

The authors declare that the research was conducted in the absence of any commercial or financial relationships that could be construed as a potential conflict of interest.

## Publisher’s note

All claims expressed in this article are solely those of the authors and do not necessarily represent those of their affiliated organizations, or those of the publisher, the editors and the reviewers. Any product that may be evaluated in this article, or claim that may be made by its manufacturer, is not guaranteed or endorsed by the publisher.
