# Supplementary material for: Azacitidine and donor lymphocyte infusion for patients with relapsed acute myeloid leukemia and myelodysplastic syndromes after allogeneic hematopoietic stem cell transplantation: A meta-analysis
Source: Front Oncol. 2022 Aug 5;12:949534. doi: 10.3389/fonc.2022.949534 (PMC9389555; doi:10.3389/fonc.2022.949534)
Supplement: Supplementary file 1 [file Presentation_1.pdf]

Supplementary Text

Terms of search strategy:

"((AML) OR (acute myelocytic leukemia) OR (acute myelogenous leukemia) OR (MDS) OR (myelodysplastic syndrome) OR (myelodysplastic syndromes)) AND ((relapse) OR (recurrence)) AND ((transplantation) OR (transplant) OR (allograft) OR (HSCT) OR (allo-HSCT)) AND ((azacitidine) OR (azacytidine)) AND ((DLI) OR (donor lymphocyte infusion) OR (donor lymphocyte infusions))"
