# Supplementary material for: Azacitidine and donor lymphocyte infusion for patients with relapsed acute myeloid leukemia and myelodysplastic syndromes after allogeneic hematopoietic stem cell transplantation: A meta-analysis
Source: Front Oncol. 2022 Aug 5;12:949534. doi: 10.3389/fonc.2022.949534 (PMC9389555; doi:10.3389/fonc.2022.949534)
Supplement: Supplementary file 2 [file Table_1.pdf]

Supplementary Table 1

Assessment of article quality by MINORS

Source	1	2	3	4	5	6	7	8	QS
Aydin et al. (2021)	2	1	1	1	0	2	2	0	9
Claiborne et al. (2019)	2	1	1	2	0	2	2	0	10
Craddock et al. (2016)	2	2	1	2	0	2	2	0	11
Czibere et al. (2010)	2	2	1	2	0	2	2	0	11
Liberatore et al. (2020)	2	1	1	1	0	2	2	0	9
Lübbert et al. (2010)	2	2	1	2	0	2	2	0	11
Martinez-Cibrian et al. (2017)	2	1	1	1	0	2	2	0	9
Poiré et al. (2021)	2	2	1	2	0	2	2	0	11
Rautenberg et al. (2020)	2	2	2	2	0	2	2	0	12
Schroeder et al. (2013)	2	2	2	2	0	2	2	0	12
Schroeder et al. (2015)	2	2	2	2	0	2	2	0	12
Steinmann et al. (2015)	2	2	1	2	0	2	2	0	11
Woo et al. (2017)	2	2	2	1	0	2	2	0	11

MINORS, methodological index for non-randomized studies
