# Supplementary material for: Azacitidine and donor lymphocyte infusion for patients with relapsed acute myeloid leukemia and myelodysplastic syndromes after allogeneic hematopoietic stem cell transplantation: A meta-analysis
Source: Front Oncol. 2022 Aug 5;12:949534. doi: 10.3389/fonc.2022.949534 (PMC9389555; doi:10.3389/fonc.2022.949534)
Supplement: Supplementary file 3 [file Table_2.pdf]

Supplementary Table 2

Supplementary Table 2.1.
Pooled CR and 2-year OS by different proportion of DLI

**Porp. of DLI administration**	**CR rate (95% CI)**	**Interaction *p***	**2-year OS (95% CI)**	**Interaction *p***
≤70%	0.293 (0.218–0.381)	0.080	0.320 (0.272–0.372)	0.223
>70%	0.194 (0.131–0.277)		0.250 (0.167–0.356)	

Supplementary Table 2.2.
Comparison between the whole studies and 4 studies with 100% proportion of DLI

**Group**	**CR rate (95% CI)**	**Interaction *p***	**2-year OS (95% CI)**	**Interaction *p***
The whole studies	0.212 (0.159–0.276)	0.590	0.310 (0.271–0.353)	0.603
4 studies with 100% proportion of DLI	0.180 (0.102–0.297)		0.357 (0.204–0.546)	
